# Dural Venous Sinus Variations Observed in Magnetic Resonance Venography at a Tertiary Care Hospital: An Observational Study

**DOI:** 10.31729/jnma.8795

**Published:** 2024-11-30

**Authors:** Sharma Paudel, Ramswarth Sah, Rakesh Kumar Singh, Prakash Kayastha, Shailendra Katwal

**Affiliations:** 1 Department of Radiology and Imaging, Tribhuvan University Teaching Hospital, Maharajgunj, Kathmandu, Nepal; 2 Department of Radiology and Imaging, Pokhara Academy of Health Sciences, Lekhnath, Pokhara, Nepal; 3 Department of Radiology and Imaging, National Trauma Center, Mahankal, Kathmandu, Nepal

**Keywords:** *anatomical variations*, *dural sinuses*, *magnetic resonance venography*

## Abstract

**Introduction:**

The dural venous system, composed of various sinuses, plays a crucial role in draining deoxygenated blood from the central nervous system. Understanding its anatomical variations is essential to differentiate it from pathological conditions like cerebral venous sinus thrombosis. This study aims to evaluate the anatomical variations of the dural venous sinuses using Magnetic Resonance Venography.

**Methods:**

An observational, cross-sectional study was performed in the Department of Radiology from September 2023 to March 2024 after the approval by the Institutional Review Committee (Reference number: 149/080/081(6-11)E2). Magnetic Resonance Venography of 109 adult patients was performed using a 1.5 Tesla MRI scanner. Diameters of the superior sagittal sinus, straight sinus, and transverse sinuses were measured. Variations in transverse sinuses, straight sinus, and confluence were categorized and analyzed.

**Results:**

The study included 52 (47.70%) male and 57 (52.30%) female, with median age of 42 (IQR: 30.5 - 56) years. The superior sagittal sinus had the diameter of 6.4 ± 1 mm. Symmetric transverse sinus variant seen was in 80 (73.39%) patients, and left transverse sinus hypoplasia was seen in 20 (18.35%) patients. Variations in the straight sinus and confluence were also documented with a true confluence (type III) was seen in 71 (65.10%) patients.

**Conclusions:**

Anatomical variations of the dural venous sinuses as observed in 1.5 Tesla MRI was quite common.

## INTRODUCTION

The dural venous system comprises paired and unpaired venous structures that collect deoxygenated blood from the central nervous system, directing it to the internal jugular veins before it enters the heart.^1^ Complexity of dural venous system and numerous anatomical variations observed, demand careful differentiation of pathological conditions like cerebral venous sinus thrombosis and septic thrombophlebitis to prevent misdiagnosis which may lead to severe neurological outcomes.^2-4^ Visualization of small veins like inferior sagittal sinus, basal veins of Rosenthal and other veins demands higher Tesla (1.5T or greater) Magnetic Resonance Imaging (MRI). Phase contrast Magnetic Resonance Venography (MRV) is particularly beneficial as it does not require intravenous contrast.^2,5^ The anatomical variations has not been studied with 1.5T or higher MRI in Nepal. Threfore, this study will provide useful information on frequency of dural venous sinus variations.

## METHODS

This observational, cross-section study was conducted in the Department of Radiology and Imaging at Tribhuvan University, Teaching Hospital. The duration of this study was six months from September 2023 to March 2024. The population of the study was patient undergoing MRI brain scans. All patients above 18 years of age were included in the study. Patients pre-diagnosed with Dural venous sinus diseases, those with any cranial pathological lesions or masses compressing or obstructing venous sinuses, and cases with motion artifacts that degraded image quality were excluded.

Sample size was calculated by finite population correction using the formula: n = N / {1+N (e)^2^}. Where, n is the sample size, N is the expected study population size during six months period, and e is the level of precision.^6^ Supposing the expected sample population size, N = 149 (total number of expected MRV brain for six month period on the basis of previous record) and p = 0.5, the calculated sample was 108.56 with 5% precision at 95% confidence level. Thus, 109 samples were included in the study. Convenience sampling was done to obtain required sample size.

Ethical approval was obtained form Institutinal Review Committee (Reference number: 149/080/081(6-11) E2). After obtaining informed consent, patients were instructed to remove all metallic objects and wear hospital gowns. Patients were also checked for any surgically implanted devices. All MRV brain scans were performed using a 1.5 Tesla Magnetom Amira Siemens MRI scanner. Patients were positioned headfirst in the supine position on the MRI table, and scanning was performed using the routine protocol and parameters established in the department (Coil: 16 channel dedicated head coil; Sequence: 3D Phase Contrast based MR venography; Velocity encoding: 10 cm/s, Frequency FOV: 220 mm; Phase FOV: 100% of frequency FOV, Phase encoding direction: Anterior to posterior; Number of slabs: 1; Slices per slab: 120; Slice thickness: 1.2 mm; Distance factor: 20% of slice thickness; Number of signal averages: 1; Concatenations: 1; Time of Repetition: ~79 mS; Time of echo: ~ 9.9 mS; Flip angle: 15 degrees; Parallel Acquisition Technique: Generalized autocalibrating partially parallel acquisitions (GRAPPA) with acceleration factor of 3; Receiver bandwidth: 320 Hz/ pixel. After completing the scans, all images meeting the inclusion criteria were randomly reviewed on the workstation. The MRV images without dural venous sinus pathologies and free from motion artifacts were selected for evaluation. For image analysis, a maximum intensity projection (MIP) image was obtained by postprocessing the source image. The diameters of the superior sagittal sinus, straight sinus, and transverse sinuses were measured at a 1 cm distance from the confluence on the MIP images. Visual inspection of the MIP images was conducted to identify normal anatomical variants. A Self-structured checklist was used as a data collection tool during the study.

Variations in transverse sinuses were divided into five categories: ^7^

0 - Symmetric (right and left transverse sinus)

1 - left hypoplastic

2 - right hypoplastic

3 - left aplastic

4 - right aplastic

Variations in straight sinus was divided into four categories: ^7^

0 - Straight sinus draining into confluence

1 - Straight sinus draining into left Transverse Sinus(TS)

2 - Straight sinus draining into right TS

3 - Straight sinus dividing into two branches and draining into their respective sides

Variations in confluence were divided into three categories.^7^

0 - type I (Superior sagittal sinus drains into one transverse sinus and the straight sinus drains into the other, with no connection between the two)

1 - type II (Superior sagittal sinus and the Straight sinus fork, and forks from both the sinuses join to form the transverse sinus)

2 - type III (A true confluence of Sinuses)

Data obtained were entered in IBM SPSS Statistics, version 26 (IBM Corp., Armonk, N.Y,. USA.and statistical analysis was carried out. Mean or median value and standard deviation or interquartile range (IQR) were used for descriptive statistics and categorical variables were presented as percentage.

## RESULTS

Among 109 patients, 52 (47.70%) were male and 57 (52.30%) were female. The age of participants ranged from 18 years to 86 years with median age of 42 (IQR 25.50 - 30.50) years. The diameter of superior sagittal sinus was 6.4 ± 1 mm, and that of the right transverse sinus was 5.90 ± 1 mm (Table 1). Symmetric transverse sinus was seen in 80 (73.39%) individuals, hypoplasia of the left transverse sinus was seen in 20 (18.35%) individuals (Table 2).

**Table 1:** Diameter of major dural venous sinuses in MRV, expressed in mean±SD (n=109).

VS	Overall (n=109)	Male (n=52)	Female (n=57)
SSS	(6.40±1) mm	(6.50±1.10) mm	(6.30±0.90) mm
SS	(3±0.80) mm	(3.10±0.60) mm	(2.9±0.8) mm
LTS	(5.10±1.30) mm	(5.00±1.40) mm	(5.20±1.27) mm
RTS	(5.90±1) mm	(5.90±1.10) mm	(6±0.90) mm

MRV=Magnetic Resonance Venography, VS=Venous Sinus, SSS= Superior Sagittal Sinus, SS=Straight Sinus, LTS=Left Transverse Sinus, RTS=Right Transverse Sinus

**Table 2:** Variation of transverse sinuses in MRV (n=109).

TS Variation	Male n (%)	Female n (%)	Total n (%)
Symmetric TS	34 (31.19)	46 (42.2)	80 (73.39)
Left TS Hypoplasia	10 (9.17)	10 (9.17)	20 (18.35)
Right TS Hypoplasia	4 (3.67)	1 (0.92)	5 (4.59)
Left TS Aplasia	3 (2.75)	-	3 (2.75)
Right TS Aplasia	1 (0.92)	-	1 (0.92)

MRV = Magnetic Resonance Venography, TS=Transverse Sinus

Straight sinus was seen draining into the confluence in 71 (65.14%) individuals, and into the left transverse sinus in 20 (18.35%) individuals (Table 3).

**Table 3:** Variation in straight sinus in MRV (n=109).

SS Variations	Male n (%)	Female n (%)	Total n (%)
SS Draining into Confluence	33 (30.28)	38 (34.86)	71 (65.14)
SS dividing into two branches and draining into their respective TS	5 (4.59)	4 (3.67)	9 (8.26)
SS draining into Left TS	10 (9.17)	10 (9.17)	20 (18.35)
SS Draining into Right TS	4 (3.67)	5 (4.59)	9 (8.26)

MRV=Magnetic Resonance Venography , TS=Transverse Sinus, SS=Straight Sinus

True confluence (type III) was seen in 71 (65.14%) individuals (Table 4).

**Table 4:** Variation of confluence of sinuses MRV (n =109).

Variation in Confluence	Male n(%)	Female n(%)	Total n(%)
Type I	2 (1.83)	7 (6.42)	9 (8.26)
Type II	13 (11.93)	16 (14.68)	29 (26.61)
Type III	37 (33.94)	34 (31.19)	71 (65.14)

MRV=Magnetic Resonance Venography

Occipital vein was present in 15 (13.8%) individuals, among which 7 (46.6%) were female and 8 (53.3%) were male.

## DISCUSSION

The measurement and variations of dural venous sinuses were studied in 109 participants in 1.5T MR system using 3D phase contrast study. Understanding these variations aids in the proper diagnosis of variant anatomy for sinus thrombosis and pre-surgical evaluations of sinuses.

Superior sagittal sinus was the largest sinus with diameter of 6.4 ± 1 mm followed by right transverse sinus (5.9 ± 1 mm) and left transverse sinus (5.1 ± 1.3 mm). Deniz et al. conducted a comparative study measuring dural venous sinus diameters using 3D SPGR-MRV and PC-MRV and found the mean diameters of the superior sagittal sinus, straight sinus, left transverse sinus, and right transverse sinus to be 7.1 mm, 4 mm, 5.2 mm, and 6 mm, respectively, which closely align with our findings.^8^ They also concluded that the left transverse sinus diameter was smaller than the right, supporting our study's results. The diameters observed in our study closely match those from a morphometric evaluation of sinus diameters via MRV in a Bangladeshi population by Yasmin et al.^9^ This suggests that variations in vessel diameter might be due to differences in head habitus among individuals included in the studies. Knowledge of these sinus diameters is important to differentiate normal sinus from partially recanalized sinus thrombosis where caliber of the sinus is reduced with normal flow in the lumen.

A symmetric transverse sinus was seen in 80 (73.4%) individuals. Symmetric transverse sinus was the most common followed by left transverse sinus hypoplasia, right transverse sinus hypoplasia, left transverse sinus aplasia, and Right transverse sinus aplasia, last two not seen in female. Alper et al. identified asymmetry in the transverse sinus, noting non-visualization or discontinuity in 24% of cases.^10^ Compared to previous studies, our study had the least frequent occurrence of aplastic transverse sinus at 3.7%. Fofi et al. studied transverse sinus morphology in patients with chronic migraine and observed such variations in 9.6% of their study population.^11^ Another study done by Chang et al. found non-visualization of either transverse sinus in 29% of cases without venous sinus thrombosis which is much higher than our study.^12^ Study conducted by Goyal et al. showed high prevalence of hypoplastic left transverse sinus in male compared female, contrary to our study which showed equal prevalence in both genders.^3^ In line with our study, Bayarogullari et al. found that hypoplastic left transverse sinuses were more common than hypoplastic right transverse sinuses, with prevalence of 49.09% and 17.53%, respectively.^7^ Transverse sinus aplasia was more in this study, left transverse sinus hypoplasia more in male. Retrospective study conducted by Jakhar et al. utilizing 3D phase contrast MRV and contrast enhanced MRV found transverse sinus asymmetry and left side hypoplasia in 24.9% of female and 19.3% of male (p=0.009). Atresia of anterior one-third of superior sagittal sinus was found to be most common variant of Superior sagittal sinus. Phase contrast was confirmed to be good option for patients with contrast allergy or renal insufficiency with comparable results to contrast based MRV.^13^ No any case of atresia of superior sigmoid sinus was found in our study. Knowledge of sinus aplasia or hypoplasia is very important as they may be mistaken for complete or partial occlusion due to sinus thrombosis.

Straight sinus was seen draining into the confluence in 71 (65.1 %) individuals, and into the left transverse sinus in 20 (18.3 %) individuals. In a study by Widjaja E et al. in 46% individuals, straight sinus was draining into the confluence and in 34% into left transverse sinus.^14^ Though the drainage pattern was similar to our study, drainage into the confluence was less. Drainage pattern of straight sinus may be important where thrombosis of one of the transverse sinus occurs.

True confluence (type III) was seen in 71 (65.1%) individuals. This is in contrast to the study of Fukusumi et al. who observed a true confluence, where all four sinuses joined to form the torcular Herophili, in only 20% of cases.^15^

Persistent occipital sinus was observed in 13.8% in our study which was significantly lower than that seen in other studies. In the study conducted by Widjaja et al.^14^ on the pediatric population, the occipital sinus was present in 18%. Persistent occipital sinuses were recognized in 57.5% of the subjects in the study performed by Fukusumi et al. The occipital sinus is worth mentioning when reporting posterior fossa masses or conditions that will require a posterior fossa craniotomy, as the sinus may be large or, more importantly, off midline and injured during the surgery.

The sample size in this study was not large enough to generalize the prevalence of variations in dural venous sinuses and their diameters in the Nepali population. Manual measurements may have introduced errors. Additionally, certain variations, such as an ectatic sinus with larger flow momentum causing the flow vortex to produce greater vorticity adjacent to solid boundaries, which were hemodynamically significant, were not included in this study, thus, became limitations of this study.

## CONCLUSIONS

This study provided insights into the normal diameter and variations of dural venous sinuses. The superior sagital sinus was the largest sinus, most of the transverse sinus were symmetric, and straight sinus was seen draining into the confluences in most of the individuals. The true confluence (Type III) was observed in most of the patients.
